# Phosphorus balance and use efficiency on 21 intensive grass-based dairy farms in the South of Ireland

**DOI:** 10.1017/S0021859614000641

**Published:** 2014-09-02

**Authors:** E. MIHAILESCU, P. N. C. MURPHY, W. RYAN, I. A. CASEY, J. HUMPHREYS

**Affiliations:** 1 Animal and Grassland Research and Innovation Centre, Teagasc, Moorepark, Fermoy, Co. Cork, Ireland; 2 Department of Chemical and Life Sciences, Waterford Institute of Technology, Cork road, Waterford, Co. Waterford, Ireland; 3 School of Agriculture and Food Science, University College Dublin, Belfield, Dublin 4, Ireland

## Abstract

Given the finite nature of global phosphorus (P) resources, there is an increasing concern about balancing agronomic and environmental impacts from P usage on dairy farms. Data from a 3-year (2009–2011) survey were used to assess farm-gate P balances and P use efficiency (PUE) on 21 intensive grass-based dairy farms operating under the good agricultural practice (GAP) regulations in Ireland. Mean stocking rate (SR) was 2·06 livestock units (LU)/ha, mean P surplus was 5·09 kg/ha, or 0·004 kg P/kg milk solids (MS), and mean PUE was 0·70. Phosphorus imports were dominated by inorganic fertilizer (7·61 kg P/ha) and feeds (7·62 kg P/ha), while exports were dominated by milk (6·66 kg P/ha) and livestock (5·10 kg P/ha). Comparison to similar studies carried out before the introduction of the GAP regulations in 2006 indicated that P surplus, both per ha and per kg MS, has significantly decreased (by 74 and 81%, respectively) and PUE increased (by 48%), mostly due to decreased inorganic fertilizer P import and improvements in P management. There has been a notable shift towards spring application of organic manures, indicating improved awareness of the fertilizer value of organic manures and good compliance with the GAP regulations regarding fertilizer application timing. These results suggested a positive impact of the GAP regulations on dairy farm P surplus and PUE, indicating an improvement in both environmental and economic sustainability of dairy production through improved resource use efficiencies. Such improvements will be necessary to achieve national targets of improved water quality and increased dairy production. Results suggest that optimizing fertilizer and feed P imports combined with improved on-farm P recycling are the most effective way to increase PUE. Equally, continued monitoring of soil test P (STP) and P management will be necessary to ensure that adequate soil P fertility is maintained. Mean P surplus was lower and PUE was much higher than the overall mean surplus (15·92 kg P/ha) and PUE (0·47) from three studies of continental and English dairy farms, largely due to the low import system that is more typical in Ireland, with seasonal milk production (compact spring calving), low use of imported feeds and high use of grazed grass.

## INTRODUCTION

Given the finite nature of global phosphorus (P) resources and the need to reduce P losses to the environment (Cordell *et al.* 2011; Huhtanen *et al.* 2011; Simpson *et al.* 2011), there is great concern for efficient P use in intensive farming systems. Irish dairy production systems tend to be relatively intensively managed compared to other Irish grassland agricultural production systems and are pasture-based, with the objective of producing milk in a low-cost system through maximizing the proportion of grazed grass in the cows’ diet (Shalloo *et al*. 2004; McCarthy *et al.* 2007; Ryan *et al.* 2011). Increasing the proportion of grazed grass reduces milk production costs and can increase the profitability of grass-based milk production systems in Ireland and other temperate climates (Dillon *et al.* 2005; Dillon 2011). Phosphorus imports, in the form of concentrate feeds and fertilizers, are key drivers of increased herbage yields and saleable milk export on most dairy farms (Aarts 2003; Spears *et al.* 2003; Gourley *et al*. 2012). More precisely, chemical P fertilizers contribute to increases in herbage yield because they supply P in a readily available form for plant uptake, which enhances root development (Lynch & Caffrey 1997) and photosynthesis (Alexander *et al.* 2008). These improved processes impact positively on overall development of grass plants and, therefore, herbage yields. However, P imports typically exceed P in milk and livestock exported off the farms (Van Keulen *et al.* 2000). This imbalance results in surplus P that is either accumulated in soil or lost from the dairy farms (Arriaga *et al*. 2009; Gourley *et al*. 2010).

Farm-gate P surplus is commonly used as an environmental indicator for the risk of P losses to the environment (Swensson 2003; Huhtanen *et al.* 2011; Weaver & Wong 2011). Even if surplus P does not predict the actual losses and loss pathways, it is a long-term risk indicator of P losses (Jarvis & Aarts 2000). However, unlike nitrogen (N) surpluses which are seen, necessarily, as an economic waste and potential environmental problem, P surpluses may be necessary, for a period of time, on farms where an increase in soil P content is required to achieve agronomic optimal soil P (Culleton *et al.* 1999) without posing a risk to the environment, if managed correctly. Surplus P potentially accumulates in the soil (Gourley *et al.* 2010), building soil fertility, or is lost in eroded material containing particulate P or P adsorbed on to organic-rich clay soil fractions (Kurz *et al*. 2005) or in soluble forms through leaching (Heathwaite 1997) or runoff. Grass-based farms can be sources of diffuse P losses (Kiely *et al.* 2007), because, by fertilizing grassland with chemical and organic fertilizers, high concentrations of potentially mobile P (PMP) are placed at or near the soil surface, where it may be susceptible to mobilization and transport to water bodies (Herlihy *et al*. 2004). These P losses can have negative environmental impacts such as eutrophication of surface waters (Clenaghan *et al.* 2005) and pollution of groundwater aquifers (Heathwaite 1997). In Ireland, P is the major limiting nutrient in surface fresh waters and increased additions may result in algal blooming (McGarrigle 2009). Losses of P also incur economic costs in two ways; the expenditure of wasted N and P inputs, at farm level, and the expenditure of clean-up associated with pollution caused as a result of such losses, more typically at regional to national levels (Buckley & Carney 2013). It has been emphasized that dairy production should be achieved in a sustainable manner, without impairing natural capital (soils, water, biodiversity) (Goodland 1997). Therefore, in the current study, P surplus, as an indicator of potential for P losses, which can be associated with environmental implications, is referred to as an indicator of environmental sustainability. In addition, due to the economic implications of these losses, P surplus is also referred to as an indicator of economic sustainability (i.e. farms’ ability to generate sufficient funds to sustain their production potential in the long run; European Commission 2001) in the current study.

Nutrient use efficiencies indicate farms’ resource use and related management decisions and are therefore considered as an indicator of farms’ agronomic performance (Halberg 1999; Oenema *et al.* 2003; Gourley *et al.* 2012). However, due to the potential economic implications of P that is not used on farms (Buckley & Carney 2013), in the current study, P use efficiency (PUE) is also considered as an indicator of economic sustainability, along with P surplus. Hence, improved nutrient use efficiency has a significant role to play in the development of more sustainable dairy production systems (Goulding *et al.* 2008). The PUE (proportion of P imports recovered in agricultural exports (Aarts 2003)) in dairy production systems is highly variable. For example, in Europe, PUE values of between 0·37 and 0·85 have been recorded (Mounsey *et al.* 1998; Van Keulen *et al*. 2000; Steinshamn *et al*. 2004; Nielsen & Kristensen 2005; Raison *et al.* 2006; Huhtanen *et al*. 2011).

Irish dairy production systems benefit from mild winters (5·1°C in January) and annual rainfall between 800 and 1200 mm, allowing grass growth all year around and an extended grazing season that can be as long as February to November (Humphreys *et al.* 2009), varying with location and soil type. Irish dairy farms are unique in Europe in that the majority operate a seasonal milk production system with compact spring calving (from January to April) so that milk production matches grass growth. The proportion of grazed grass in the diet of dairy stock is hence maximized (Humphreys *et al.* 2009), allowing for the maximum amount of milk to be produced from grazed grass and reducing requirements for feeding concentrate post-calving (Dillon *et al.* 1995). For these reasons, the potential for more effective use of P on-farm and management strategies to achieve improved PUE may be expected to differ from those of the year-round feed-based dairy production systems more typical of continental Europe and Britain (excluding Northern Ireland). In grass-based dairy production systems, there are a number of factors affecting PUE, such as soil P-sorption capacity in relation to soil P inputs, uneven dispersal of excreta leading to uneven soil P content (in grazing enterprises), the ability of grass plants to convert P from applied chemical P fertilizer and manure into biomass in herbage, utilization by animals of grass herbage grown and the biological potential of cows to convert P from concentrate feeds and herbage into milk (Gourley *et al.* 2010). More effective use of P imports in concentrate feeds and fertilizer P, and soil P resources, can potentially contribute to decreased imports and increased PUE (Nielsen & Kristensen 2005; Huhtanen *et al.* 2011).

The on-going debate over P supply and demand together with the concern for water quality affected by P lost from agricultural land supports the need to ensure that P is used efficiently on farms (Pieterse *et al*. 2003; Syers *et al.* 2008; Simpson *et al.* 2011; Weaver & Wong 2011). In the EU, the Water Framework Directive (WFD) (European Communities 2000) was introduced with the objective of protecting and improving the quality of groundwater and surface water bodies. In Ireland, the WFD was first implemented as the Water Policy Regulations (European Communities 2003), in 2003. To ensure water quality, these regulations established a concentration limit of 0·03 mg molybdate reactive phosphorus (MRP)/l or 35*μ*g/l PO_4_ (European Communities 2009). Additionally, the Nitrates Directive (91/676/EEC) (European Council 1991) has established guidelines in relation to farming practices to reduce nitrate (NO_3_) leaching that are implemented in each member state through a National Action Programme (NAP). In Ireland, these are legislated as the good agricultural practice (GAP) regulations (European Communities 2010), first passed in 2006 (European Communities 2006). The GAP regulations establish farming practices to reduce NO_3_ leaching but also limit P use on farms and establish soil P indices. Under the Regulations, farms are limited to a stocking rate (SR) of 170 kg organic N/ha, equivalent to 2 livestock units (LU)/ha, or 2 dairy cows/ha. The Regulations also establish the quantity of available P that can be applied to grass and other crops (depending on factors such as SR, soil test P (STP) and crop type), the volume of slurry storage required (depending on factors such as location, local rainfall and stock type and number), closed periods in winter months during which spreading of organic and inorganic fertilizers is restricted (depending on location in the country) and other restrictions on spreading based on soil conditions, topography, weather and distance to water features.

The GAP regulations established a P index system for grassland soils based on STP. Index 1 (0·0–3·0 mg P/litre (l)) and 2 (3·1–5·0 mg P/l) soils are considered deficient in P and require a build-up of soil P to reach agronomic optimum. The target index is 3 (5·1–8·0 mg P/l), at which the soil is considered to have optimum P to meet crop demand without having negative impacts on the environment (Ryan & Finn 1976; Herlihy *et al.* 2004; Power *et al.* 2005). Soils within index 4 (>8 mg P/l), with high P status, are considered in excess of agronomic optimum and at greater risk of P loss to water. The new index system involved the lowering of the upper limits previously advised for grassland soils: from 6 to 5 mg P/l for index 2, and from 10 to 8 mg P/l for index 3. The aim was to reduce P losses from grassland while maintaining agricultural production (M. Treacy, personal communication). Soil P status is assessed every 5 years on Irish farms (European Communities 2010). For SRs up to 2 LU/ha, the maximum P fertilizer application allowed ranges between 39 kg/ha for soils in index 1 to 0 kg/ha for soils in index 4 (European Communities 2010).

The GAP measures are intended to increase PUE and retention of N and P within the production systems and minimize losses from farms to water. However, most of the existing data on dairy farm P balances in Ireland date from the period before the implementation of the Regulations in 2006 (Mounsey *et al.* 1998; Ruane *et al*. 2013). There is no study on farm-gate P balance on Irish dairy production systems after the implementation of GAP regulations and, in the European context, very few farm-gate P balances on grassland-based dairy farms (e.g. Van Keulen *et al*. 2000; Aarts 2003; Swensson 2003; Nielsen & Kristensen 2005; Gamer & Zeddies 2006; Raison *et al.* 2006). Steinshamn *et al*. (2004) and Huhtanen *et al.* (2011) examined P balances and use efficiencies in dairy production systems but these were based on modelling and experimental studies.

Therefore, the objectives of the current study were: (i) to assess farm-gate P balances and use efficiencies on 21 commercial intensive dairy farms operating under the GAP Regulations in Ireland and compare these to pre-Regulations studies to investigate the impact of the Regulations; (ii) to identify the factors influencing PUE on these farms; and (iii) to explore potential approaches to increase PUE and decrease P surpluses on these farms. For this purpose, data on P imports and exports were recorded on 21 dairy farms participating in the INTERREG-funded DAIRYMAN project over 3 years, from 2009 to 2011.

## MATERIALS AND METHODS

### Farm selection and data collection

Twenty-one commercial intensive dairy farms were selected, located in the South of Ireland, in counties Cork, Limerick, Waterford, Tipperary, Kilkenny and Wicklow. These farms were pilot farms involved in the INTERREG-funded DAIRYMAN project (www.interregdairyman.eu) focusing on improving resource use efficiency on dairy farms in Northwest Europe. Farm selection was based on the likely accuracy of data recording, eight of the farms in the current study having been involved in a previous similar study (GREENDAIRY; Ruane *et al.* 2013), and all the farmers being willing to provide data. The selected farms were known as being progressive in their approach to farm management and, therefore, may not be fully representative of all Irish dairy farms. However, the farm area, SR and milk yield per cow indicated that the participating farms were close to, but slightly above, the national average for dairy farms. Grass-based milk production from spring calving cows was the main enterprise on all the selected farms.

Key farm characteristics are given in Table 1. Mean total utilized agricultural area (TUAA) was 71 (s.d.=24·8) ha, mean SR was 2·06 (s.d.=0·32) LU/ha, and mean milk yield was 5308 (s.d.=464) litres/cow between 2009 and 2011. For comparison, national mean values for dairy farms were 52 ha for TUAA, 1·90 LU/ha for SR, and 4956 litres/cow for milk yield, during the same timeframe (Connolly *et al*. 2009; Hennessy *et al.* 2010, 2011). Seventeen of the farms in the current study participated in the Rural Environment Protection Scheme (REPS). This is a programme co-funded by the EU and the Irish government whereby farmers are rewarded financially for operating to a set of guidelines consistent with an agri-environmental plan drawn up by an approved planning agency (DAFM 2004). Important conditions for receiving REPS financial support were to limit SR to 2 LU/ha and to apply chemical fertilizers to the farming area according to fertilizer plans drawn up for their farms (DAFM 2004).

**Table 1.** Mean values (and standard deviation) for total utilized agricultural area (with crop area in brackets), annual mean temperature, annual rainfall, soil test phosphorus, pH, stocking rate, milk yields, concentrate feeds, and estimated harvested grass through grazing and silage; soil type for 21 Irish dairy farms between 2009 and 2011

Farm	TUAA (crops) (ha)	Temp. (°C)	Rainfall (mm/year)	Soil type	STP (mg/l)	pH	SR (LU/ha)	Milk yield (l/cow)	Conc (kg DM/LU)	Grass (kg DM/LU)
1	85	9·6	1077	CL	6	5·9	2·2	5319	268	4139
2	67	9·8	1124	C	4	6·4	2·4	6010	499	4169
3	73	9·8	1124	C	9	6·5	2·1	5688	221	4304
4	50	10·1	1373	L	7	6·5	2·7	5309	571	3691
5	74 (1·2)	10·1	1373	L	7	5·7	1·8	5149	611	3891
6	63 (3·9)	10·1	1373	L	3	5·3	1·9	5672	568	3632
7	47	9·6	1077	L	2	5·6	2·4	5080	471	3922
8	58	10·1	1373	C	7	5·9	2·5	5671	580	4033
9	51	9·6	1077	C	6	5·9	2·0	5431	466	4089
10	130 (5·5)	10·1	1373	L	7	5·7	2·0	5207	394	3898
11	40	10·1	1373	L	4	5·3	2·4	4229	615	3508
12	52	10·1	1373	L	8	6·0	1·8	5613	604	3886
13	81	9·6	1077	C	8	5·8	1·8	5290	710	3730
14	96 (6·7)	9·8	1124	SL	5	6·0	1·8	4415	302	3472
15	128	9·8	1124	L	5	6·2	1·9	4671	484	3858
16	78 (13·4)	10·2	1453	C	7	6·5	1·6	6038	801	3746
17	72	9·6	1077	C	6	6·2	2·5	4928	463	4002
18	48	9·8	1124	CL	4	6·0	1·9	5549	732	3567
19	71 (2·3)	9·8	1124	C	7	6·2	2·2	5500	251	2919
20	76 (6·2)	10·1	1373	SL	9	5·8	2·0	5174	265	4011
21	48 (1·6)	10·1	1373	L	3	5·6	1·4	5522	386	4108
Mean	71 (5·6)	9·9	1235	–	6	5·9	2·1	5308	488	3837
s.d.	24·8 (3·91)	0·22	145	–	1·9	0·35	0·32	464	166	309

TUAA, total utilized agricultural area; temp., temperature; CL, clay-loam; L, loam; C, clay; SL, sandy-loam; STP, soil test phosphorus; SR, stocking rate; LU, livestock units; l, litres; conc., concentrate feeds; DM, dry matter; s.d., standard deviation.

On the selected farms, data were collected on a monthly basis between 2010 and 2011 and included grassland area, area under crops, type of crops and proportion of crops fed to livestock, livestock numbers and type of livestock, number of days spent grazing, and imports of manure, concentrate feeds, bedding material, silage, chemical P fertilizers and other agro-chemicals, as well as exports of milk, manure, crops and silage. For chemical P fertilizers, amounts imported onto farms as well as the amounts applied to land were recorded on a monthly basis. For 2009, similar data were obtained from farm records and farm advisors. Data collected for the 3 years were cross-checked with secondary data sources such as Single Farm Payment forms (data forms required from farmers for participation in state schemes) (DAFM 2010*a*). Data on livestock imports and exports were extracted from the Dairy Management Information System (DAIRYMIS) (Crosse 1991). Values for amounts of milk sold off the farms were extracted from the reports on milk deliveries coming from the cooperatives supplied by the farmers. Data on soil types were extracted from REPS forms for the participating farms and from the national soil survey (Gardiner & Radford 1980) for the remainder. Data on mean annual rainfall and temperature were extracted from an Irish Meteorological Service database for different weather stations located in, or close to, the area of study, at Cork airport, Roche's point, Gurteen, Johnstown Castle and Oak Park (Irish Meteorological Service 2013).

The annual amount of pasture harvested and utilized on-farm through grazing and silage on each farm was modelled using the Grass Calculator (Teagasc 2011) based on the difference between the net energy (NE) provided by imported feeds (concentrates and forages) and the NE requirements of animals for maintenance, milk production and body weight change (Jarrige 1989). It was assumed that 1 kg dry matter (DM) of grass equals 1 unit of feed for lactation (UFL).

Stocking rate was expressed as LU per ha for TUAA. One dairy cow was considered equivalent to 1 LU and 1 bovine less than 1 year old equivalent to 0·3 LU (Connolly *et al*. 2009).

### Soil sampling and analysis

Eleven soil samples, on average, were taken per farm on one occasion during the study period, the farmers being required to sample their farms at least once every 5 years (European Communities 2010). Samples were taken using a standard soil corer (50 mm diameter), sampling to a depth of 100 mm. Each sample area was ≤4 ha, with sample areas evenly distributed across each of the farms. The sample areas were also carefully selected to ensure areas used for grazing and silage production were both represented. At least 50 soil cores were taken from each sample area, in a zigzag pattern. Care was taken to avoid unusual spots in the sample area, such as old fences, ditches and around gateways and feed troughs (M. Treacy, personal communication). Each sample was carefully mixed, before smaller representative bulked samples were extracted and sent for analysis to Teagasc Johnstown Castle Research Centre. Samples were analysed for soil pH and Morgan's Soil P concentrations using the standard laboratory procedures for Ireland, as described by Byrne (1979). Soil samples were dried for 16 h at 40°C in a forced draught oven with moisture extraction. Soil pH was determined by mixing 10 ml of dried sieved (2 mm) soil with 20 ml of H_2_O and, after being allowed to stand for 10 min, measuring the pH of the suspension using a digital pH meter with glass and calomel electrodes. For soil P concentrations, soil samples were extracted in a one part soil to five parts solution ratio with a 10 g sodium acetate solution buffered at pH 4·8 (Morgan's solution). Six millilitres (ml) of dried soil was extracted with 30 ml of Morgan's solution using a Brunswick Gyratory shaker for 30 min at constant temperature (20°C). The suspension was then filtered using No. 2 Whatman filter paper. Analysis for P content was then carried out on the clear extract by spectrophotometry (M. Treacy, personal communication). The same sampling procedure and soil analyses were used for two similar previous studies (Mounsey *et al.* 1998; Ruane *et al*. 2013), which the current study was compared to.

### Farm-gate phosphorus balances and phosphorus use efficiencies

Phosphorus imports and exports were calculated both on a monthly and an annual basis. Phosphorus in chemical fertilizer was calculated by taking into account the P content of fertilizers applied to land. Monthly imported amounts of concentrate feeds and forages were assumed to be exhausted by the end of each month. Due to the fact that P content of imported concentrates and forages onto farms was not directly measured, it was assumed to be 5 kg P/tonne (t) of concentrate and forage (European Communities 2010).

Phosphorus in livestock imported on, or exported off, the farms was calculated by using standard values for live weight (Ruane *et al*. 2013) and multiplying it by 0·01 (McDonald *et al.* 1995). Phosphorus in exported milk was calculated by considering a P content of 0·0009 kg P/kg of milk (McDonald *et al.* 1995).

The farm-gate P balance was calculated as the difference between total P import and total P export (Weaver & Wong 2011) and was expressed on both an area basis (kg P/ha) and a unit product basis (kg P/kg milk solids (MS)) (Fangueiro *et al*. 2008) for years 2009–2011. Phosphorus use efficiency was calculated as the ratio between total P export and total P import, expressed as a proportion (Huhtanen *et al*. 2011) for years 2009–2011.

The same principles for calculating P inputs, outputs, balances and PUE were followed in two similar previous studies (Mounsey *et al.* 1998; Ruane *et al*. 2013), which the current study was compared to.

### Statistical analysis

Descriptive statistics were applied using SPSS Inc. 17.0 to calculate means and standard errors (George & Mallery 2008). Normal distribution of residuals was tested using Shapiro–Wilk, with values <0·05 indicating abnormal distribution. Log transformation was required to ensure homogenity of variance (Tunney *et al*. 2010) for some of the variables. Therefore, TUAA, milk fat and protein concentration, P imports per ha from fertilizer P, feeds and livestock, total P import, milk P export, P balance per ha and per kg MS, PUE, P imports per kg MS from fertilizer P and feeds, MS exports per cow, comparative STP values, P imports from fertilizers and feeds, P exports in sold milk, P balance per ha and per kg MS and PUE in the current study, and the studies of Ruane *et al.* (2013) and Mounsey *et al.* (1998) were transformed using a log10 base (*y*=log10(*x*)).

Differences in mean STP, TUAA, SR, milk yields, milk protein and fat concentration, concentrate feed imports, P imports, P exports, P balance per ha and per kg MS and PUE between years and farms were analysed using repeated measures analysis of variance (ANOVA). A significance level of 0·05 or less (0·01 and 0·001) indicated statistically significant differences among the means. A significance level of 0·05 or higher indicated a 95 or higher per cent of certainty that the differences among the means are not the result of random chance (George & Mallery 2008). Such results were presented as not significant (NS).

The statistical models included farm and year effects on each of the tested variables. The 21 farms were considered as replicates. The models used were:

1. *Y*_*i*_=*μ*+*a*_*i*_+*e*_*i*_, where *Y*_*i*_ is the tested variable, *a*_*i*_ is the effect of *i*th farm (*i*=1,….,21), and *e*_*i*_ is thethe residual error term; and
2. *Y*_*i*_=*μ*+*b*_*j*_+e_*i*_, where *Y*_*i*_ is the tested variable, *b*_*j*_ is the effect of *j*th year (*j*=2009, 2010, 2011), and *e*_*i*_ is the residual error term.

Multiple stepwise linear regression was undertaken to investigate relationships between key dependent and independent variables presented in Table 2. The choice of the statistical models was dependent on the potential significance of independent variables and their potential impact on the dependent variables. Non-significant independent variables were automatically removed from the models (Table 2). The probability for acceptance of new terms (*F*) was 0·10 (Groot *et al*. 2006) and the confidence interval was 0·95. All relationships between variables were assessed for outliers, normality and colinearity.

**Table 2.** Investigated and significant multiple stepwise linear regression models

Investigated	Significant
LgFrtP=*μ*+*β*LgTUAA+*β*STP+*β*SR+*β*MSE+*β*GD+*σ*_est_	LgFrtP=*μ*−STP+*σ*_est_
LgFdP=*μ*+*β*SR+*β*MSE+*β*GD+*σ*_est_	LgFdP=*μ*+*β*SR−*β*GD+*σ*_est_
LgMP=*μ*+*β*SR+*β*MSE+*β*GD+*β*LgFrtP+*β*LgFdP+*σ*_est_	LgMP=*μ*+SR+*σ*_est_
LP=*μ*+*β*SR+*β*GD+*β*LgFrtP+*β*LgFdP+*σ*_est_	NS
LgPbal=*μ*+*β*STP+*β*SR+*β*MSE+*β*GD+*β*LgFrtP+*β*LgFdP+*σ*_est_	NS
LgPUE=*μ*+*β*SR+*β*MSE+*β*GD+*β*LgFrtP+*β*LgFdP+*σ*_est_	LgPUE=*μ*−*β*LgFrtP−*β*LgFdP+*σ*_est_
LgPMS=*μ*+*β*LgMS+*β*GD+*β*LgFrtPMS+*β*LgFdMS+*σ*_est_	LgPMS=*μ*−*β*LgMS+*σ*_est_

LgFrtP, log-transformed chemical fertilizer P applied to land; LgFdP, log-transformed feeds phosphorus (P) import; LgMP, log-transformed milk P export; LP, livestock P export; LgPbal, log-transformed P balance per ha; LgPUE, log-transformed P use efficiency; LgPMS, log-transformed surplus P per kg milk solids; LgTUAA, log-transformed total utilized agricultural area; STP, soil test P; SR, stocking rate; MSE, milk solids export per ha; GD, number of grazing days; LgMS, log-transformed milk solids export per cow; LgFrtPMS, log-transformed chemical fertilizer P applied to land per kg milk solids; LgFdMS, log-transformed feeds P import per kg milk solids; *β*=standardized coefficient of regression; *σ*_est_, standard error of the estimate; NS, not significant.

Uncertainty analysis was carried out by calculating the coefficient of variation as the ratio between standard deviation and mean values (Gourley *et al.* 2010) for each P import, P export, P balance and PUE on the 21 farms between 2009 and 2011, expressed as a proportion.

## RESULTS

### Phosphorus imports

**Fig. 1.**
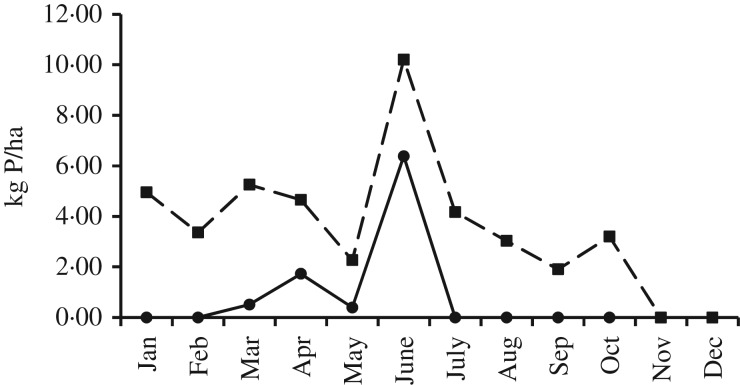
Monthly application rates of chemical **(-●-)** and organic **(- -■- -)** P fertilizers (kg P/ha) on 21 Irish dairy farms between 2009 and 2011.

There was a high degree of variation in mean P imports between years and farms (Table 3). Mean total P import was 16·85 kg P/ha (Table 3). There were significant differences in mean total P import between farms, ranging from 3·64 to 26·94 kg/ha over the 3 years (Table 3). The coefficient of variation for mean total P import between farms was 0·39 over the 3 years. There were also significant (*P*<0·01) differences in mean total P import between years, ranging from 15·21 to 19·99 kg/ha (Table 3). The main sources of P import onto farms were imported feeds and chemical fertilizers, accounting for around 0·50, each, of total P import. Mean P import from feeds was 7·62 kg P/ha (Table 3). There were no significant differences in mean P import from feeds between farms (Table 3), but there were significant differences (*P*<0·001) in mean P import from feeds between years, ranging from 4·69 to 11·13 kg/ha (Table 3). Mean fertilizer P import was 7·61 kg P/ha (Table 3) and there were significant differences (*P*<0·01) between farms, ranging from 1·69 to 20·15 kg/ha over the 3 years (Table 3). The coefficient of variation for mean fertilizer P import between farms was 0·64 over the 3 years. There were no significant differences in mean fertilizer P import between years (Table 3). On a monthly basis, mean chemical fertilizer P applied to land was the highest between April and June, at 2·83 (s.d.=3·14) kg P/ha (Fig. 1).

**Table 3.** Mean values (and standard errors), grand means between years and ranges between farms for chemical P fertilizers applied to land, P imports in feedstuffs and livestock, P exports in sold milk and livestock, farm-gate P balances, P use efficiencies per ha and P balance per kg milk solids for 21 Irish dairy farms between 2009 and 2011; standard error of the means for transformed data in brackets; P-values from ANOVA are included

	Year			Grand mean	s.e.m.	Range farms	*P*-value	
	2009	2010	2011				Y	F
P imports (kg P/ha)								
Chemical fertilizers applied	8·43	7·91	6·50	7·61	0·78 (0·054)	1·69–20·15	NS	<0·01
Feeds	4·69	11·13	7·04	7·62	0·60 (0·033)	2·52–13·44	<0·001	NS
Livestock	2·24	0·95	1·67	1·61	0·13 (0·041)	0·06–4·62	NS	NS
Total	15·36	19·99	15·21	16·85	1·04 (0·032)	3·64–26·94	<0·01	<0·05
P exports (kg P/ha)								
Milk	6·22	7·22	6·56	6·66	0·20 (0·013)	4·27–9·52	NS	<0·001
Livestock	4·46	5·52	5·32	5·10	0·277	2·63–9·43	NS	<0·01
Total	10·68	12·74	11·88	11·76	0·412	7·44–17·45	NS	<0·001
P balance (kg P/ha) (kg P/ha)	4·68	7·25	3·33	5·09	1·07 (0·067)	−7·42 to 19·48	<0·05	0·01
P use efficiency	0·69	0·63	0·78	0·70	0·10 (0·034)	0·30–1·58	NS	0·01
P balance (kg P/kg MS)	0·0004	0·011	0·003	0·004	0·001 (0·0629)	−0·01 to 0·03	NS	NS

P, phosphorus; MS, milk solids; S.E.M., standard error of the means; Y, year; F, farm; NS, not significant.

There was a significant negative relationship (*R*^2^=0·21; *P*<0·05) between mean log-transformed chemical fertilizer P applied to land and STP (*β*=−0·46). An increase of 0·34 mg/l in mean STP was associated with a decrease of 0·03 (0·92, not transformed) kg/ha in mean log-transformed chemical fertilizer P applied to land.

There was a significant relationship (*R*^2^=0·20; *P*<0·01) between mean log-transformed feed P import and mean SR (*β*=0·34) and mean number of days spent grazing (*β*=−0·24). An increase of 0·07 LU/ha in mean SR was associated with an increase of 0·02 (0·55, not transformed) kg/ha in mean log-transformed feed P import. An increase of 2·20 days/year in mean number of days spent grazing was associated with a decrease of 0·02 (0·55, not transformed) kg/ha in mean log-transformed feed P import.

### Phosphorus exports

There was a high degree of variation in mean P exports between farms (Table 3). Mean total P export was 11·76 kg P/ha (Table 3) and while there were no significant differences in mean total P export between years, there were significant differences (*P*<0·001) in mean total P export between farms, ranging from 7·44 to 17·45 kg/ha over the 3 years (Table 3). The coefficient of variation for mean total P export between farms was 0·24 over the 3 years (Table 3). The main sources of P export were sold milk and livestock, accounting for 0·56 and 0·44, respectively, of total P export. Mean milk P export was 6·66 kg P/ha (Table 3), with significant differences (*P*<0·001) seen in mean milk P export between farms, ranging from 4·27 to 9·52 kg/ha over the 3 years (Table 3). The coefficient of variation for mean milk P export between farms was 0·21 over the 3 years. However, there were no significant differences in mean milk P export between years (Table 3). Mean livestock P export was 5·10 kg P/ha (Table 3) and significant differences (*P*<0·01) in mean livestock P export were seen between farms, ranging from 2·63 to 9·43 kg/ha over the 3 years (Table 3). The coefficient of variation for mean livestock P export between farms was 0·32 over the 3 years and there were no significant differences in mean livestock P export between years (Table 3).

There was a significant positive relationship (*R*^2^=0·45; *P*<0·001) between mean log-transformed milk P export and mean SR (*β*=0·67). An increase of 0·07 LU/ha in mean SR was associated with an increase of 0·008 (0·26, not transformed) kg/ha in mean log-transformed milk P export.

There was no significant relationship between livestock P export and mean SR, number of days spent grazing, log-transformed chemical fertilizer P applied to land or log-transformed feed P import (Table 2).

### Phosphorus balance and phosphorus use efficiency

There was a P deficit on eight farms and a P surplus on 13 farms. Mean P balance (P imports less P exports) was 5·09 kg P/ha (Table 3). There were significant differences (*P*<0·01) in mean P balance between farms, ranging from −7·42 to +19·48 kg/ha over the 3 years (Table 3). The coefficient of variation for mean P balance between farms was 1 over the 3 years. There were also significant differences (*P*<0·05) in mean P balance between years, ranging from 3·33 to 7·25 kg/ha in 2010 (Table 3). Mean PUE (P exports divided by P imports) was 0·70 (Table 3). There were significant differences (*P*<0·01) in mean PUE between farms, ranging from 0·30 to 1·58 over the 3 years (Table 3) and the coefficient of variation for mean PUE between farms was 0·40 over the 3 years. There were no significant differences in mean PUE between years (Table 3). Mean P balance per kg MS was 0·004 kg P/kg MS (Table 3) and there were no significant differences in mean P balance per kg MS between farms and years (Table 3).

There was a significant negative relationship (*R*^2^=0·71; *P*<0·001) between mean log-transformed PUE and mean log-transformed chemical fertilizer P applied to land (*β*=−0·75) and mean log-transformed feed P import (*β*=−0·30). An increase of 0·03 (0·92, not transformed) kg/ha in mean log-transformed chemical fertilizer P applied to land and of 0·02 (0·55, not transformed) kg P/ha in mean log-transformed feed P import was associated with a decrease of 0·03 (0·13, not transformed) in mean log-transformed PUE.

There was a significant negative relationship (*R*^2^=0·20; *P*<0·01) between mean log-transformed P balance per kg MS and mean log-transformed MS export per cow (*β*=−0·45). An increase of 0·02 (13, not transformed) kg MS/cow in mean log-transformed MS export per cow was associated with a decrease of 0·05 (0·003, not transformed) kg P/kg MS in mean log-transformed P balance per kg MS.

There was no significant relationship between P balance per ha and mean STP, SR, MS export, number of days spent grazing, log-transformed chemical fertilizer P applied to land and log-transformed feed P import (Table 2).

## DISCUSSION

### Phosphorus imports, exports, balances and use efficiencies

Total P import, export and surplus in the current study were close to, but slightly above, the national average for dairy farms and PUE was slightly lower than the national average found by Buckley *et al.* (2013) (mean total P import of 13 kg P/ha, mean total P export of 8·9 kg P/ha, mean P surplus of 4·1 kg P/ha, and mean PUE of 0·83). This would suggest that results from the current study may be taken as indicative of the national situation. However, caution must be taken in this regard due to the relatively low number of farms involved (21).

The overall coefficient of variation (0·54) for P imports, exports and balances and PUE, was within the range reported in other studies on farm-gate nutrient balances (0·64, Mounsey *et al.* 1998; 0·51, Nielsen & Kristensen 2005; 0·48, Ruane *et al*. 2013).

### Factors affecting phosphorus balances and use efficiencies across farms

Differences in mean chemical P fertilizers applied to land per ha between farms were principally associated with differences in mean STP. Mean STP content varied between 2·29 and 8·99 mg/l between farms. For the scope of the current study (assessment of farm-gate P balances on dairy farms operating under GAP regulations), the relationship between chemical fertilizer P applied to land and soil P status was investigated to illustrate the extent to which the farmers complied with the GAP regulations imposing higher P fertilization rates for soils with low P status and lower P fertilization rates on soils with higher soil P status; the compliance with GAP regulations in terms of P fertilization rates is one reason explaining the chemical fertilizer P imports and the actual P application to land. The results showed differences between recommended amounts of chemical P fertilizers, in line with GAP regulations, and the actual amounts of P applied to land. More precisely, in the fertilizer plans, the recommended chemical fertilizer P application rates ranged between 0 and 37·50 kg P/ha, the higher rates corresponding to farms with a higher proportion of Index 1 and 2 soils. In practice, P fertilizer application rates, averaged across the farm area, ranged between 1·69 and 20·15 kg P/ha between farms. The actual values and the negative relationship between mean chemical fertilizer P applied to land and mean STP indicate compliance with recommended fertilization rates and the GAP regulations. The difference between the recommended and actual P fertilization rates indicates that farmers with high P soils are relying more on soil P reserves to support herbage yields, and are not fully replacing P being removed in herbage. The actual P fertilization rates were lower than the rates between 14 and 40 kg P/ha, which can be taken up by pastures in one growing season, in Ireland (Ryan & Finn 1976; Power *et al.* 2005). Of course, there are also P inputs to pastures from imported feeds and recycling to soil of P taken up in the sward. This trend will save money on inputs in the short term and can be expected to reduce the proportion of high P (Index 4) soils, reducing the risk of P loss to water, as was intended in the GAP regulations. At the same time, it will be necessary to monitor soil P contents and P application rates to ensure adequate soil fertility is maintained in the future (Lalor *et al*. 2010). The fact that STP explained only 0·21 of the variation in mean chemical fertilizer P applied to land indicates that a number of other factors are important, such as use of organic P fertilizers, concentrate P imports (which affects the overall farm chemical fertilizer P allowance under the GAP regulations), economic considerations, weather and grass growth conditions, advisory impact and understanding and planning on the part of the farmer, for example.

The significant positive relationship between feed P import and SR suggests increased requirement for feed imports to support higher SRs. Concentrate feed imports per animal varied significantly between farms, from 221 to 801 kg DM/LU. These imports were probably determined by harvested grass, ranging between an estimated 2919 and 4304 kg DM/LU and targeted milk yields per cow, ranging between 4229 and 6038 litres/cow. Targeted milk yields per cow were included in development plans introduced in 2009 for each farm by farm advisors. One of the goals in the development plans was increased milk yield per cow by amounts ranging between 100 and 400 litres/cow between 2009 and 2011. The decrease in feed P import with number of days grazing suggests that extending the grazing season is an effective strategy to decrease feed P import, by increasing the proportion of grazed grass in the diet. The fact that SR and days grazing explained only 0·20 of the variation in feed P import suggests that other factors are important, such as advisory impact, economic and environmental factors.

The significant positive relationships between milk P export per ha and SR implies that increasing SR is an effective strategy to increase milk P export. Furthermore, this could decrease P surplus and increase PUE, because P in sold milk was the main form of exporting P off the farms. However, from 16·85 kg P/ha of mean total P import, only 6·66 kg P/ha or 0·39, on average, was exported in sold milk, meaning that the impact of milk P export on P surplus and PUE was rather low. The P content of sold milk is very unlikely to increase, and therefore there is a need to optimize the use of P imports, principally feed, and on-farm P resources relative to P exports in milk, to decrease P surplus and increase PUE. It is also notable that livestock exports accounted for a large proportion of P exports and there may also be scope to improve P balances and PUE here.

The fact that PUE decreased principally with chemical fertilizer P applied to land but also feed P import, explaining 0·71 of the variation in PUE, suggests that decreasing fertilizer P and feed P imports may be the most effective strategy to increase PUE. The remainder of the variation in PUE could be attributed to factors such as differences in soil P status relative to the agronomic optimum (between 5·1 and 8·0 mg P/l; Ryan & Finn 1976; Herlihy *et al.* 2004; Power *et al.* 2005) and farm-specific efficiency of P recycling and P losses between soil, pasture, animals and milk and livestock for export (Spears *et al.* 2003). It is important to note that agronomic optimal P management in grassland aims to achieve target soil P contents and may operate at a surplus for a number of years to build up soil P to optimal values. While the effective uptake zone of plants’ roots can be extended by associated mycorrhizae (Caldwell *et al.* 1985) and plants may use other mechanisms to mobilize soil P in P-deficient soils, the levels of STP considered as optimal have been established through grassland field trials in Ireland (Ryan & Finn 1976; Herlihy *et al.* 2004; Power *et al.* 2005) and are, therefore, considered appropriate.

A decrease in fertilizer and feed P imports combined with improved on-farm P recycling may increase PUE. Improved nutrient recycling on farms is consistent with one of the targets in the Food Harvest 2020 national strategy for sustainable growth of the agricultural sector (DAFM 2010*b*). On a global scale, increases in PUE over the long term, along with P recovery and reuse from all waste streams throughout the food production system (from animal excreta to crop wastes) are suggested to contribute to sustainable P use (Cordell *et al.* 2011).

Results suggest that an increase in MS exports per cow can contribute to reduced P surplus per kg MS. In grazed grass-based production systems, increased MS production and exports per cow may be achievable with low fertilizer and feed P use by optimizing other management aspects such as grazing management, grass utilization (O'Donovan *et al.* 2002; Kennedy *et al.* 2005) and management of herd genetic potential (Berry *et al.* 2007). On the other hand, an increase in MS production per cow can lead to increased P surplus per ha and potentially higher P losses, if it is not achieved in an efficient manner.

### Factors affecting phosphorus balances and use efficiencies across years

Phosphorus feed P imports and P surplus per ha were greater in 2010 compared with 2009 and 2011. The increased feed P imports were probably to support a SR that was 0·18 LU/ha greater than 2009 and 0·19 LU/ha greater than 2011. The higher SR in 2010 was associated with higher feed imports, both in kg per ha and in kg per LU, and with higher milk yields per cow, of 5411 litres/cow in 2010 compared with 5120 litres/cow in 2009 and 5291 litres/cow in 2011. This equates to a response of 2·40 litres milk/kg DM of additional feeds compared with 2009 and 0·69 litres milk/kg DM compared with 2011. A similar response in milk production, of 1·06 kg/cow per additional kg of imported feeds, was reported by Shalloo *et al.* (2004).

The increase in mean feed P import in 2010 contributed to increased mean total P import, which was 4·63 kg P/ha greater compared with 2009 and 4·78 kg P/ha greater compared with 2011. The increased total P import resulted in an increase in P surplus (7·25 kg P/ha) of 36% compared with 2009, and 55% compared with 2011. Others have found similar results (Smith *et al.* 2003). The principle reason would appear to be reductions in PUE associated with the increase in feeds P imports. These results highlight the necessity of assessing balances and use efficiencies in aggregate over a number of years.

### Phosphorus balance and use efficiency before and after the good agricultural practice regulations

The results of the current study were compared with similar studies, completed between 2003 and 2006 (Ruane *et al*. 2013) and in 1997 (Mounsey *et al.* 1998), before the introduction of the GAP regulations, to investigate possible impacts of these Regulations on P balances and PUE on Irish dairy farms. The study of Ruane *et al.* (2013) was carried out on 21 intensive dairy farms, of which eight were also involved in the current study, whereas the study of Mounsey *et al.* (1998) was on 12 intensive dairy farms. However, these intensive farms had SRs of 2·37 LU/ha (Ruane *et al*. 2013) and 2·58 LU/ha (Mounsey *et al.* 1998), respectively, compared with the national average SR of 1·85 LU/ha in 2005/06 (Connolly *et al*. 2006, 2007) and 1·47 LU/ha in 1997 (Fingleton 1997). Therefore, they may not be fully representative of all Irish dairy farms. Also, the farms in those studies were stocked more intensively than the mean SR of 2·06 LU/ha in the current study.

Mean P surplus was significantly lower (*P*<0·001) in the current study, at 5·09 kg P/ha, than Ruane *et al.* (2013) (5·61 kg P/ha) and Mounsey *et al.* (1998) (19·50 kg P/ha), whereas PUE was significantly higher (*P*<0·001), at 0·70, than Ruane *et al.* (2013) (0·68) and Mounsey *et al.* (1998) (0·37). Similarly, mean P surplus per kg MS was significantly lower (*P*<0·01), at 0·004 kg P/kg MS, compared to Ruane *et al.* (2013) (0·017 kg P/ha) and Mounsey *et al.* (1998) (0·021 kg P/ha). Results suggest a trend for decreased P surplus per ha and per kg MS, and improved PUE on Irish dairy farms over the period covered by these studies (1997–2011) and following the introduction of the GAP regulations in 2006, associated with a trend for decreasing stocking density. This trend would have both agronomic and environmental implications. From an agronomic perspective, it will be necessary to monitor soil P to ensure adequate soil fertility for sward growth (Lalor *et al.* 2011). From an environmental perspective, this should lead to less potential for P loss from the system.

There are a number of factors determining these differences between the three studies. The first factor was a significantly lower (*P*<0·001) mean SR in the current study, of 2·06 LU/ha, in comparison with 2·37 LU/ha in Ruane *et al.* (2013) and 2·58 LU/ha in Mounsey *et al.* (1998). The lower SR in the current study had further impacts on chemical P fertilizer applied to land and milk and livestock P exports.

The second factor was a significantly lower (*P*<0·001) mean chemical fertilizer P applied to land, of 7·61 kg P/ha, in the current study, compared with 10·22 kg P/ha in Ruane *et al.* (2013) and 23·45 kg P/ha in Mounsey *et al.* (1998). It would seem likely that this decrease was due to improved awareness of management of soil P status on farms (Lalor *et al.* 2010) and GAPs in P management such as more appropriate rates of application and better use of on-farm organic P fertilizers, as introduced in the GAP regulations.

The third factor differing between the studies suggests that this was indeed the case, as 0·42 of annual organic fertilizer P (farm yard manure and slurry) was applied between mid-January and April in the current study, compared with 0·55 in Ruane *et al.* (2013) but only 0·14 in Mounsey *et al.* (1998). There was no application of organic fertilizers after October in the current study and in Ruane *et al.* (2013), whereas in Mounsey *et al.* (1998), 0·31 was applied between November and January. This significant shift in the timing and proportion of organic P fertilizer application is consistent with advice on best practice indicating better fertilizer replacement value for spring application (Alexander *et al.* 2008) and with the GAP regulations (European Communities 2010) that prohibit application of organic fertilizers during the ‘closed period’, from mid-October to mid/end January. Also, spring application of organic P, besides reducing the requirement for imports of inorganic P, coincides with the development phase of grass plants and, therefore, can improve PUE in grasslands (Alexander *et al.* 2008). The concurrent decrease in chemical fertilizer P use indicates an improved awareness of the fertilizer value of organic manures and accounting for them in nutrient management planning. This was illustrated in Fig. 1, which indicates the appreciation of on-farm organic sources of P, and also presents challenges in terms of the ability of farmers to target P, as there is more uncertainty in application rates for organic P fertilizers, and the ability to apply it can be more limited spatially and temporally in comparison with the chemical P fertilizers.

The farms in the current study had a significantly lower (*P*<0·001) mean STP content of 5·64 mg/l compared to Ruane *et al.* (2013) (8·20 mg/l) and Mounsey *et al.* (1998) (11·68 mg/l). This is in line with the historical variation in STP in agricultural soils, with an increase from *c*. 1 mg/l in the early 1950s to 9 mg/l in the 1990s (Tunney 1990), and a decrease to 6·7 mg/l in 2003 (Bourke *et al.* 2008) and from 7·3 to 4·0 mg/l between 2007 and 2011 (Wall *et al.* 2012). In the current study, the implementation of GAP regulations obliged the farmers to operate STP contents considered optimal for response in herbage yields, of between 5·10 and 8·00 mg/l (European Communities 2010). The fact that the farms in the current study were operating at lower STP combined with lower surpluses and higher PUEs than the previous studies suggests much more efficient P cycling with much less potential to lose P to water.

**Table 4.** Comparative mean values (and standard errors) for total utilized agricultural area, stocking rate, national average stocking rate, soil test P, milk yield, milk protein and fat concentration, concentrate feed, chemical P fertilizers applied to land, imports of P in feedstuffs, and livestock, exports of P in milk and livestock, farm-gate P balances per ha, P use efficiencies, and P balance per kg milk solids on dairy farms before and after the implementation of Good Agricultural Practice regulations in Ireland; standard error of the means for transformed data in brackets; P-values from ANOVA are included

	Current study	Ruane *et al.* (2013)	Mounsey *et al.* (1998)	s.e.m.	*P*-value
TUAA (ha)	71	59	65	3·3 (0·02)	NS
Stocking rate (LU/ha)	2·06	2·37	2·58	0·049	<0·001
National stocking rate (LU/ha)	1·90	1·85	1·47	–	–
STP (mg/l)	5·64	8·20	11·68	0·46 (0·025)	<0·001
Milk yield (l/cow)	5308	5167	5588	65·4	NS
Milk protein (%)	3·4	3·4	3·3	0·01 (0·001)	<0·001
Milk fat (%)	4·0	3·8	3·7	0·02 (0·002)	<0·001
Concentrate feed (kg DM/LU)	488	549	480	29·4	<0·05
P imports (kg P/ha)					
Chemical fertilizer applied	7·61	10·22	23·45	1·41 (0·067)	<0·01
Feeds	7·62	7·58	7·82	0·46 (0·025)	NS
Livestock	1·61	0	0	–	–
Total	16·85	17·80	31·27	1·55 (0·036)	<0·05
P exports (kg P/ha)					
Milk	6·66	7·35	9·13	0·30 (0·016)	<0·01
Livestock	5·10	4·84	2·64	0·241	<0·001
Total	11·76	12·19	11·77	0·338	NS
P surplus (kg P/ha)	5·09	5·61	19·50	1·28 (0·084)	<0·001
P use efficiency	0·70	0·68	0·37	0·08 (0·034)	<0·001
P surplus (kg P/kg MS)	0·004	0·017	0·021	0·015 (0·0629)	<0·01

TUAA, total utilized agricultural area; LU, livestock units; STP, soil test phosphorus; l, litres; DM, dry matter; MS, milk solids; S.E.M., standard error of the means; NS, not significant.

### Phosphorus balance and use efficiency of Irish dairy farms in an international context

The results of the current study were compared with similar international studies as outlined in Table 5. In this comparison, the term ‘continental European farms’ refers to the Dutch farms in Aarts (2003), the Danish farms in Nielsen & Kristensen (2005) and the French farms in Raison *et al.* (2006).

**Table 5.** Comparative number of farms, type of system, grassland area, stocking rate, milk yield, P imports from chemical fertilizers and feedstuffs, P exports in milk, P surpluses, and P use efficiencies in different regions

Reference	Region	No. of farms	Type of system	Grassland (proportion of TUAA)	SR (LU/ha)	Milk yield (l/ha)	Fertilizer P import (kg P/ha)	Feed P import (kg P/ha)	Milk P export (kg P/ha)	P surplus (kg P/ha)	PUE
Current study	South of Ireland	21	G/C	0·93	2·06	7569	7·61	7·62	6·66	5·09	0·70
Aarts (2003)	The Netherlands	17	G/C	0·76	1·74	14 528	8·50	24·00	19·00	13·50	0·58
Nielsen & Kristensen (2005)	Denmark	25	D+A	0·59	1·54	12 631	5·00	22·00	7·00	16·00	0·46
Raison *et al.* (2006)	Scotland	10	G/C	0·94	1·60	7155	13·20	12·76	7·92	17·60	0·33
	South of Ireland	24	G/C	1·00	2·10	7757	11·00	7·04	7·48	7·92	0·62
	SW England	13	G/C	0·84	2·20	9847	13·20	11·88	9·68	15·40	0·44
	Brittany	15	G/MS	0·70	1·40	5315	4·40	17·16	5·28	15·84	0·48
	Pays de la Loire	13	G/MS	0·65	1·30	4837	5·72	10·12	4·84	9·68	0·57
	Aquitaine	9	C/MS/MG	0·39	1·20	6053	23·76	13·20	5·72	21·56	0·43
	Basque country	16	0G	0·88	2·70	15 304	10·12	45·32	16·28	39·96	0·35
	Galicia	18	0G	0·58	3·00	19 723	35·20	57·20	18·04	71·72	0·24
	North Portugal	21	0G	0·00	6·10	34 760	29·92	66·00	32·12	51·04	0·48
Gourley *et al.* (2012)	Australia	37	G/C	0·83	1·75	13 975	16·60	9·20	10·00	25·80	0·32

No., number; G/C, grazing–cutting; D+A, dairy+arable crops; G/MS, grazing-maize for silage; C/MS/MG, cutting-maize for silage-maize for grain; 0G, zero-grazing; TUAA, total utilized agricultural area; SR, stocking rate; LU, livestock units; l, litres; P, phosphorus; PUE, phosphorus use efficiency.

Chemical fertilizer P applied to land in the current study (7·61 kg P/ha) was lower than the Dutch farms in Aarts (2003) (8·50 kg P/ha), the English and Irish farms (12·46 kg P/ha) and the French farms (11·29 kg P/ha) in Raison *et al.* (2006), and the Australian farms in Gourley *et al.* (2012) (16·60 kg P/ha), but higher than the Danish farms in Nielsen & Kristensen (2005) (5·00 kg P/ha).

Feed P import in the current study (7·62 kg P/ha) was much lower compared with Aarts (2003) (24·00 kg P/ha), Nielsen & Kristensen (2005) (22·00 kg P/ha), the English and Irish farms (10·56 kg P/ha) and the French farms (13·49 kg P/ha) in Raison *et al.* (2006). The main reason for higher feed P imports in these studies was the high import/export system of dairy production that is more typical of dairy production in continental Europe, characterized by year-round milk production, high use of imported feeds, lower use of grazed grass and high milk yields per ha and per cow. In contrast, a low import/export system is more typical in Ireland, with seasonal grass-based milk production (compact spring calving), low use of imported feeds, high use of grazed grass and lower milk yields per ha and per cow. The continental European studies (14 528 litres/ha, Aarts 2003; 12 631 litres/ha, Nielsen & Kristensen 2005) and the English and Irish farms in Raison *et al.* (2006) (8253 litres/ha) had much higher milk yields per ha compared with the current study (7569 litres/ha). The French farms in Raison *et al.* (2006) had lower mean milk yield per ha (5401 litres/ha) due to mixed agricultural production (milk, maize for export) on some of the farms, lower SR (1·3 LU/ha compared with 2·06 LU/ha, in the current study) and lower milk quota (5108 litres/ha compared with 6850 litres/ha, in the current study). The higher milk yields per ha were also associated with higher mean milk P exports per ha on the Dutch farms in Aarts (2003) (19·00 kg P/ha) and the English and Irish farms in Raison *et al.* (2006) (8·36 kg P/ha) compared with the current study (6·66 kg P/ha). Despite the higher milk yields in Nielsen & Kristensen (2005), mean milk P export (7·00 kg P/ha) was similar to the current study, due to mixed agricultural production (milk, cereals for export). On the French farms in Raison *et al.* (2006), the mean milk P export (5·28 kg P/ha) was lower than in the current study, probably due to their lower milk yields and SR.

In the study of Gourley *et al.* (2012), on Australian farms, year-round grazing allowed for high use of grazed grass and therefore lower imports of feeds (9·20 kg P/ha) than the continental European farms and the English and Irish farms in Raison *et al.* (2006), but higher than the Irish farms in the current study, due to much higher milk yields per ha (13 975 litres/ha).

Despite the relatively low milk P export per ha, mean P surplus (5·09 kg P/ha) in the current study was much lower than that reported by Aarts (2003) (13·50 kg P/ha), Nielsen & Kristensen (2005) (16·00 kg P/ha), the English and Irish farms (13·64 kg P/ha) and the French farms in Raison *et al.* (2006) (15·69 kg P/ha) and the Australian farms in Gourley *et al.* (2012) (25·80 kg P/ha). This reflects the low import/export model of dairy production in Ireland. Mean PUE in the current study (0·70) is much higher than that reported by Aarts (2003) (0·58), Nielsen & Kristensen (2005) (0·46), the English and Irish farms (0·46) and the French farms (0·49) in Raison *et al.* (2006), and the Australian farms in Gourley *et al.* (2012) (0·32).

It can be concluded that Irish dairy farms tend to operate with lower feed P imports, relatively low fertilizer P imports and lower P surpluses per ha than most other European dairy farms at lower exports (litres milk/ha) and that this is largely due to the low import/export system that is more typical in Ireland with seasonal milk production (compact spring calving) (Buckley *et al.* 2000), low use of imported feeds (Dillon *et al.* 1995), high use of grazed grass (Horan 2009) and relatively low milk yields per cow (Humphreys *et al.* 2009). All other factors being equal, one might expect less P losses to the environment under conditions of lower P surplus.

## CONCLUSIONS

A survey of 21 Irish dairy farms from 2009 to 2011 found a mean P surplus of 5·09 kg/ha, or 0·004 kg P/kg MS, and a mean PUE of 0·70. Farm-gate P imports were dominated by feeds (7·62 kg P/ha) and inorganic fertilizer (7·61 kg P/ha), while exports were dominated by milk (6·66 kg P/ha) and livestock (5·10 kg P/ha). Comparison to similar studies carried out before the introduction of the GAP regulations in 2006 would suggest that P surplus, both per ha and per kg MS, have significantly decreased (by 74 and 81%, respectively) and PUE increased (by 48%) following the introduction of the GAP regulations. These improvements have mostly been achieved through decreased chemical fertilizer P applied to land and improvements in P management, with a notable shift towards spring application of organic manures, consistent with advice on best practice and with the GAP regulations that prohibit application of organic fertilizers during the ‘closed period’ from mid-October to mid/end January. A concurrent decrease in chemical fertilizer P use indicates an improved awareness of the fertilizer value of organic manures and accounting for them in nutrient management planning. The cumulative effect of the improvement in management of organic manures and the decrease in chemical fertilizers may have led to the lower mean STP values observed in the current study, closer to values considered optimal for pasture production. These results would suggest a positive impact of the GAP regulations on dairy farm P surplus, PUE and STP.

Taking surplus P per ha and STP as indicators of local environmental pressure, this indicates that the environmental sustainability of milk production has improved. Taking PUE as an indicator of agronomic performance, the improvement in PUE also indicates that agronomic performance has improved concurrently. This demonstrates that is possible to improve both environmental and economic sustainability of dairy production through improved resource use efficiencies. Such improvements will be necessary to achieve national targets of improved water quality under the EU Water Framework Directive and increased dairy production, as set out in the Food Harvest 2020 Report. Results suggest that optimizing chemical fertilizer P applied to land and feed P imports combined with improved on-farm P recycling may be the most effective way to increase PUE. Equally, continued monitoring of STP and P management will be necessary to ensure that adequate soil P fertility is maintained.

Mean P surplus was lower and mean PUE was higher than the overall mean surplus (15·92 kg P/ha) and mean PUE (0·47) from three studies of continental European dairy farms. It can be concluded that Irish dairy production systems, on average, tend to operate with lower chemical fertilizer P applied to land and feed P imports and lower P surpluses than other continental European dairy production systems and that this is largely due to the low import system that is more typical in Ireland, with seasonal milk production (compact spring calving), low use of imported feed stuffs, high use of grazed grass and lower milk yields per cow. All other factors being equal, one might expect less P losses to the environment under conditions of lower P surplus.
